# Joint shape morphogenesis precedes cavitation of the developing hip joint

**DOI:** 10.1111/joa.12143

**Published:** 2013-11-25

**Authors:** Niamh C Nowlan, James Sharpe

**Affiliations:** 1 EMBL-CRG Systems Biology Program, Centre for Genomic Regulation, UPFBarcelona, Spain; 2 Department of Bioengineering, Imperial CollegeLondon, UK; 3 Institució Catalana de Recerca i Estudis Avançats (ICREA)Barcelona, Spain

**Keywords:** 3D imaging, acetabulum, femur, joint cavitation, optical projection tomography, pelvis

## Abstract

The biology and mechanobiology of joint cavitation have undergone extensive investigation, but we have almost no understanding of the development of joint shape. Joint morphogenesis, the development of shape, has been identified as the ‘least understood aspect of joint formation’ (2005, *Birth Defects Res C Embryo Today* **75**, 237), despite the clinical relevance of shape morphogenesis to postnatal skeletal malformations such as developmental dysplasia of the hip. In this study, we characterise development of early hip joint shape in the embryonic chick using direct capture 3D imaging. Contrary to formerly held assumptions that cavitation precedes morphogenesis in joint development, we have found that the major anatomical features of the adult hip are present at Hamburger Hamilton (HH)32, a full day prior to cavitation of the joint at HH34. We also reveal that the pelvis undergoes significant changes in orientation with respect to the femur, despite the lack of a joint cavity between the rudiments. Furthermore, we have identified the appearance of the ischium and pubis several developmental stages earlier than was previously reported, illustrating the value and importance of direct capture 3D imaging.

## Introduction

The biology and mechanobiology of joint cavitation have undergone extensive investigation, but we have very little understanding of joint shape morphogenesis, which has been identified as the ‘least understood aspect of joint formation’ (Pacifici et al. 2005). The first step of joint formation occurs in the cartilage anlage, where chondrocytes at the presumptive joint site stop proliferating and become compacted to form what is called the interzone. From the interzone, the articular cartilage surfaces and the synovial cavity develop (Pacifici et al. 2005). It has been reported that once the cavity has been initiated, the surfaces of the opposing cartilage rudiments undergo gradual shape morphogenesis so that eventually the ends of the two bones form a functioning, friction-minimising contact surface, i.e. a synovial joint (Pacifici et al. 2005). No concrete mechanisms directing the development of joint shape have been discovered, despite the clinical significance of this process. Hip joint development is particularly relevant clinically, due to the fact that the most commonly presenting condition resulting from abnormal fetal joint morphogenesis is developmental dysplasia of the hip (DDH). DDH is a condition in which hip joint morphogenesis does not proceed correctly, and occurs when the juvenile hip joint is unstable, partially dislocated (subluxated) or completely dislocated (Homer et al. 2000). The condition has an incidence as high as 1 in 100 newborns (Homer et al. 2000), and if treatment is not administered or is unsuccessful, osteoarthritis is likely to develop. An enhanced understanding of the sequence of events in the morphogenesis of hip joint development in animal models is key to advancing our understanding of DDH.

The developing pelvis received a significant amount of attention around the turn of the 20th century. Bardeen & Lewis (1901) were the first to describe the early stages of pelvic and limb development in human embryos using histological analyses. However, the majority of the early studies of the developing pelvic girdle and hindlimb used the chick as a model system, with particular focus on the initiation of the pelvic girdle between embryonic days 4 and 5 (Mehnert, 1887; Johnson, 1893; Lebedinsky, 1913; cited in Pomikal & Streicher, 2010). In more recent times, Malashichev et al. (2005) examined pelvic girdle development during chick development using serial sections and whole-mount skeletal preparations. The authors observed chondrification (as measured with Alcian Blue) of the ilium at Hamburger Hamilton (HH; Hamburger & Hamilton, 1951) stage 26, the pubis at HH29 and the ischium at HH31. In another study, Malashichev et al. (2008) performed grafting and extirpation experiments in the chick to reveal that development of the pubis and ischium is regulated by ectodermal signals, while development of the ilium is regulated by both ectodermal and somatic signals. Pomikal & Streicher (2010) describe early pelvic girdle development in the mouse, using 3D models reconstructed from serial histological sections of hindlimbs between Theiler Stages (TS) 18–25. The authors found that the pelvis forms from a single mesenchymal condensation with ischial, pubic and iliac processes, detectable at TS20. At formation, the pelvic element is distant to the body axis and gradually approaches the axial skeleton through ilial elongation, which continues until late TS24 when the sacro-iliac joint is formed. The pubic and ischial processes also undergo elongation until fusion of the tips of the processes to form the foramen obturatum at late TS24. The authors describe how the pelvic element changes dramatically in orientation with respect to the body axis, from an almost perpendicular orientation at TS21 to an acute angle with respect to the body axis by TS24 (Pomikal & Streicher, 2010). Using the same approach, Pomikal et al. (2011) describe pelvic girdle development in *Rana tempoaria* (common frog), and reported similar results regarding initial formation of the pelvic girdle as a single mesenchymal condensation and its location far from the axial skeleton.

Studies involving sectioning and *post-hoc* reconstruction into 3D, such as some of those described above, can lead to distortions and lack of fine detail. Direct 3D capture using optical projection tomography (OPT; Sharpe et al. 2002) enables capture of tissue-specific markers that can be fluorescent or colorimetric and, once captured, data can be represented in a number of ways such as surface representations and virtual sections. OPT has previously been used for visualising the organisation of musculoskeletal tissues of the developing limb (DeLaurier et al. 2006, 2008; Roddy et al. 2009). Roddy et al. (2009) focussed specifically on development of the avian knee (stifle) and described how the developing joint displays the main shape characteristics of an adult joint by HH34, such as the lateral and medial condyles and the cranial cnemial crest.

The interaction between the proximal femur and pelvic girdle is critical to our understanding of developmental disorders of the hip and limb, and while a small number of studies on the pelvic girdle have been performed, no study has described the development of the interaction of the proximal femur, acetabulum and pelvic girdle that form the hip joint in 3D over critical periods of prenatal development. In this study, we characterise hip joint development in chick embryos between 5 and 10 days of incubation using direct 3D capture with OPT. We focus on the identification of key features and their changes in shape and orientation during the dynamic early stages of hip joint development, providing the first comprehensive description of hip joint morphogenesis in the chick embryo.

## Materials and methods

Fertilised eggs, supplied by a local farm (Granja Gilbert, Cataluña, Spain), were incubated at 38 °C in a humidified incubator for 6–9 days. The harvested embryos were staged using Hamburger Hamilton (HH) criteria (Hamburger & Hamilton, 1951). At least four embryos per stage between HH Stages 26 and 35 were stained for cartilage with Alcian Blue and scanned in 3D using OPT (Sharpe et al. 2002), as described previously (Nowlan et al. 2008). Surface representations of the left limbs were created using ImageJ (http://rsbweb.nih.gov/ij/, last accessed April 2013; Abràmoff et al. 2004), and the limb considered most representative of each stage was selected for further analysis. The hip joint was visualised in a number of ways using Paraview (http://www.paraview.org/, last accessed April 2013; Henderson, 2007): surface views of the pelvis and femur from the femoral posterior aspect and the pelvic ventral aspect, a virtual section through the pelvis and femur, with the section taken through the dorsal–ventral plane of the femur, surface view of the dorsal aspect of the pelvis (with the femur virtually dissected out), and the ventral and dorsal surface views of the femur with the pelvis virtually dissected. Rudiments were virtually dissected out from the full image using ImageJ. Three-dimensional models of the structure were visualised with colour-coding to highlight the different component rudiments. Histological analyses were performed for right limbs between HH30 and HH35, where hindlimbs were sectioned through the anterior–posterior plane of the femur and stained as described previously (Nowlan et al. 2010) with Weigert's Iron Hematoxylin, Fast Green and Safranin-O.

## Results

At the earliest stage examined, HH26 (about 5 days of incubation), the surface of the proximal femur is indistinguishable from the pelvis (data not shown). Therefore, detailed analyses were performed from HH27 onwards. As specimens had already been selected based on the HH (Hamburger & Hamilton, 1951) staging system, there was not a large degree of variation between replicates for the main morphological features, but there was some variation in size. The most typical rudiment from each time-point was selected for detailed display.

### Pelvic girdle development

**Figure 1:**
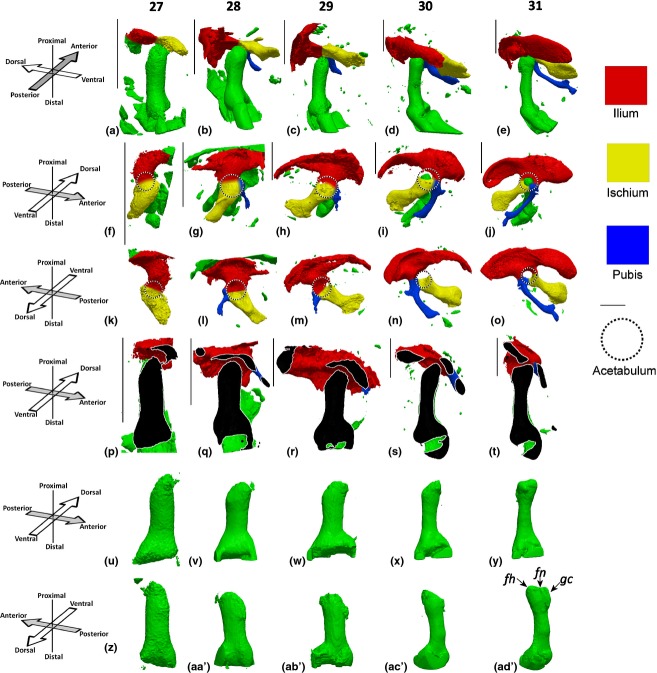
Pelvic and femoral development between HH27 and HH31. (a–e) Pelvis and femur, posterior aspect of femur; (f–j) pelvis and femur, ventral aspect of pelvis; (k–o) dorsal aspect of pelvis; (p–t) virtual section though the dorsal–ventral plane of the femur, section taken through the femoral head and parallel to the main axis of the femur; (u–y) ventral aspect of the femur, view; (z–ad′) dorsal aspect of the femur. fh, femoral head; fm, femoral neck; gc, greater trochanter. Left limbs shown, orientations for a–e and p–ad′ with respect to femur, orientations for f–o with respect to body axis. Scale bars: 1 mm.

**Figure 2:**
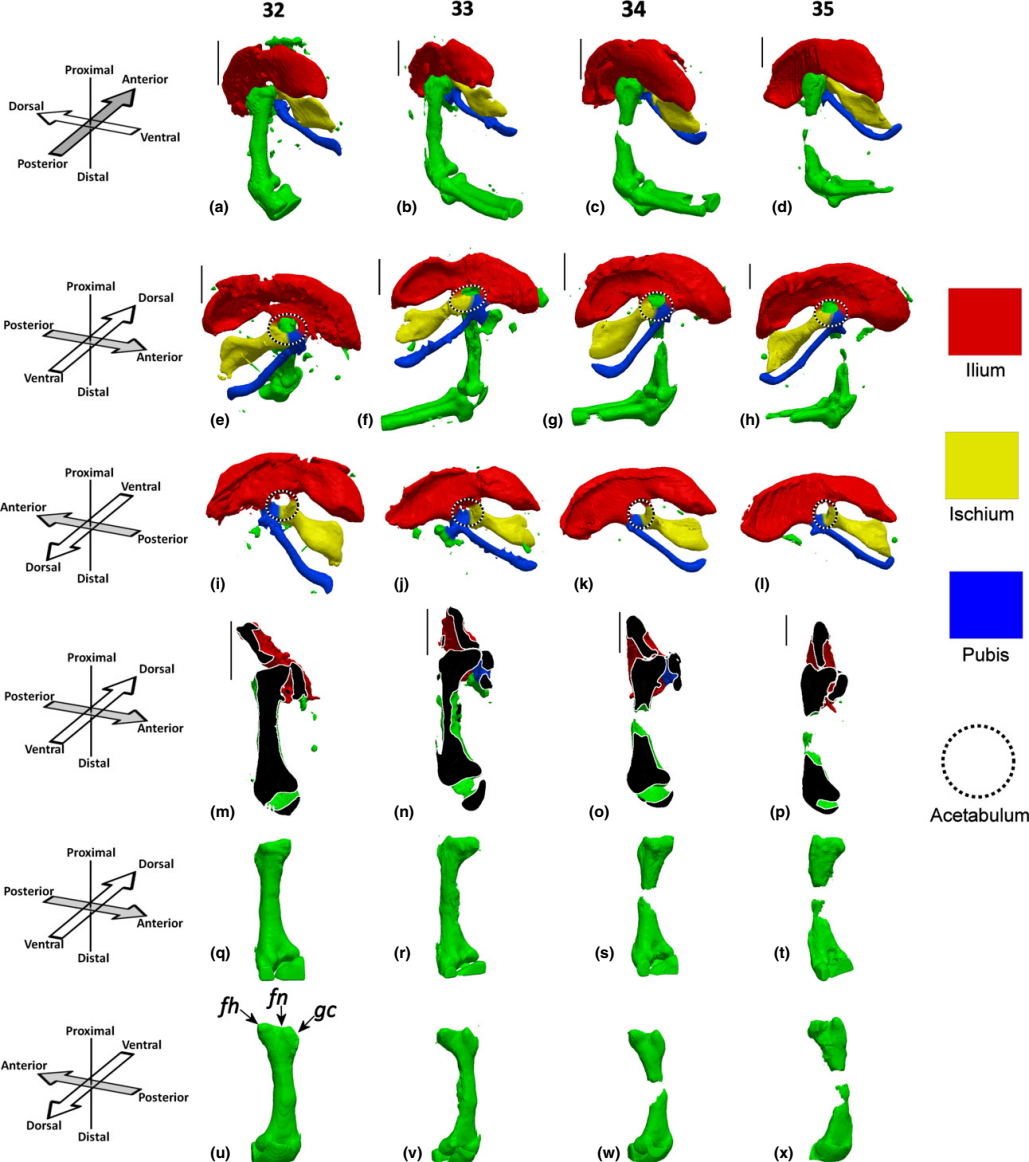
Pelvic and femoral development between HH32 and HH35. (a–d) Pelvis and femur, posterior aspect of femur; (e–h) pelvis and femur, ventral aspect of pelvis; (i–l) dorsal aspect of pelvis; (m–p) virtual section though the dorsal–ventral plane of the femur, section taken through the femoral head and parallel to the main axis of the femur; (q–t) ventral aspect of the femur, view; (u–x) dorsal aspect of the femur. fh, femoral head; fm, femoral neck; gc, greater trochanter. Left limbs shown, orientations for a–d and m–x with respect to femur, orientations for e–l with respect to body axis. Scale bars: 1 mm.

At HH27, the ilium and ischium are identifiable, with no evidence as yet of the pubis, as shown in Fig. 1a,f. A hollow above the femoral head, the future location of the acetabulum is detectable as early as HH27, as shown in Fig. 1k (circled), and visible in part encapsulating the femoral head in Fig. 1p. Initiation of the pubis is identifiable from HH28 (about 6 days of incubation), and the ischium and ilium begin to take on more refined shapes at the same stage (Fig. 1b,g,l). By HH29, the ilium is the biggest element of the pelvic girdle, and has taken on its characteristic curved shape (Fig. 1). The pubis has thickened but remains very short at this stage (Fig. 1h,m). At HH30, the acetabulum develops a cleft between the ischium and ilium, but by HH31 the elements have re-joined to form a perforated acetabulum, which is a feature of the avian skeleton (Makovicky & Zanno, 2011; Fig. 1j,o). The pubis undergoes a dramatic lengthening between HH29 and HH30, as shown in Fig. 1i,n. By HH31, the distinctive shape of the ilium has been formed, and the pubis has lengthened further and curved anteriorly to approach the ischium (Fig. 1j,o). Between HH31 and HH34, the shapes of the rudiments of the pelvic girdle do not change substantially, apart from lengthening and curvature of the pubis and a convergence of the ilium and ischium. Starting at HH33, the cartilage at the mid-diaphysis of the femur is gradually replaced by calcified cartilage through the process of endochondral ossification, leading to a loss in Alcian Blue staining in this region (Fig. 2; HH33–HH35). At HH35, the ilium and the ischium connect, with the gap between them forming the ilio-ischiatic foramen, while the pubis and ischium also form a distal connection that will later contain the obturator foramen (Fig. 2h,l).

### Shape morphogenesis of the proximal femur

At HH27, the femoral head is poorly defined, but a protrusion of the future femoral head is recognisable (Fig. 1u,z). By HH28, the femoral head remains quite rudimentary in shape, but an indication of the greater trochanter is evident in a dorsal view (Fig. 1aa′). One day later, at HH30 (about 7 days of incubation in our hands), the femoral head has become more refined, and greater outgrowth of the femoral head is evident (Fig. 1x,ac′). By HH31, the key morphological features of the femoral head, greater trochanter and femoral neck are detectable (Fig. 1y,ad′) and are even more pronounced by HH32 (Fig. 2q,u). Between HH32 and HH35, these features become more defined, but no further dramatic changes in the femoral head take place (Fig. 2r–t,v–x).

### Definition of the hip joint: relationship between the pelvis and proximal femur

Between HH27 and HH29, the main axis of the ilium and ischium in the pelvic girdle is almost perpendicular to the femur, as shown in Fig. 1a–c. However, from HH30, the pelvic girdle begins a gradual rotation with respect to the distal limb until, by HH35, the main axis of the pelvis is at an acute angle to the femur, as shown in Fig. 2a–d,m–p. The complexity of the reciprocal contact surfaces between the femur and acetabulum also starts to change dramatically at HH30. Prior to HH30, the acetabulum curves around the distal femur, as shown in Fig. 1p–r. With the perforation of the acetabulum at HH30, the three rudiments meeting at the acetabulum start to encapsulate the femoral head until, by HH34, the ilium indents into the femoral neck and the femoral head is directly in contact with the pubis (as shown in Fig. 2p) and the ischium (data not shown).

### Hip joint cavitation

**Figure 3:**
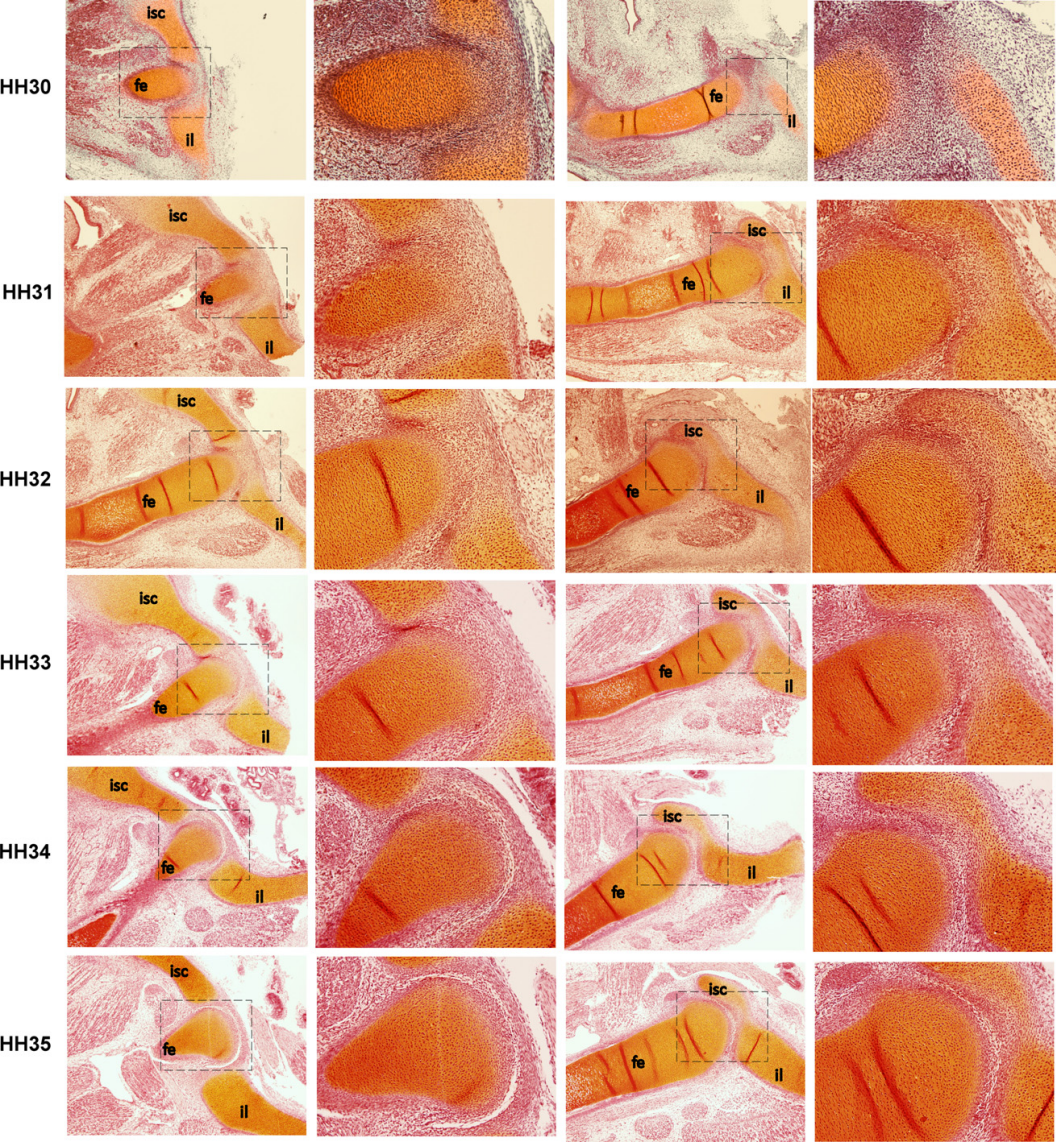
Histological sections of the right hip joint between HH30 and HH35 through the anterior–posterior plane of the femur showing that no cavity is present until HH34. First and third column, magnification × 4, second and fourth column, magnification × 10. fe, femur; il, ilium; isc, ischium. Proximal is to the right and ventral to the top.

Although 2D sections are less suitable for visualising morphology than direct capture 3D images, they are useful in confirming histological changes over time. Using traditional histological techniques, two views through the anterior–posterior plane of the femur for specimens from HH30 to HH35 were characterised. The first view shown is of a section through the acetabulum where the ilium and ischium meet, and the second a lateral section through the acetabulum, where the ilium encapsulates the femoral head, as presented in Fig. 3. The hip joint cavity, visible as a clear white line in the joint region (Fig. 3), is not present until HH34 in the chick hip joint, which is similar to cavitation time-points for other joints in the chick reported by previous studies (Osborne et al. 2002). In other words, in contrast to previous reports, we have been able to show that the major anatomical features of hip joint shape (greater trochanter, femoral head and neck, acetabulum) form and develop before cavitation. While it was challenging to obtain consistent sections through the dorsal–ventral plane, shape features (such as the greater trochanter) detected in the 3D OPT data were also observed in the histological data at the same stages (data not shown).

## Discussion

In this paper, we have described how the most important anatomical and functional features of the hip joint emerge during early chick development. To summarise, the acetabulum, ischium and ilium are present and identifiable at HH27 (about 5.5 days of development), while the pubis first becomes evident half a day later at HH28. By HH31, the femoral head, greater trochanter and femoral neck are present, and these features gradually become more defined until they are recognisable as comparable to the adult features. Also by HH31, the three elements of the pelvis have taken on their characteristic morphologies. The perforated acetabulum of the chick is present and formed by HH31, and the ilio-ischiatic foramen is present by HH35. Between HH30 and HH33, the pelvis undergoes a substantial rotation relative to the femur, until the pelvis is at an acute angle to the femur by HH35. Our histological results demonstrate that these key shape features and the change in orientation of the femur and pelvis develop prior to the complete formation of the articular cavity, which is only visible by HH34, meaning that the early stages of joint shape morphogenesis precede cavitation, in contrast to what has previously been suggested (Pacifici et al. 2005).

The use of 3D imaging has revealed novel features of pelvic girdle development that have not previously been observed using whole-mount or histological data. We have shown that the ilium and ischium are both identifiable as chondrified bodies using Alcian Blue staining from HH27, and the pubis from HH28, in contrast to a previous study that detected chondrification of the ischium at HH31 and of the pubis at HH29 (Malashichev et al. 2005). Our data demonstrate rotation of the pelvis with respect to the femur in a process that begins at HH31. While Pomikal and colleagues (Pomikal & Streicher, 2010; Pomikal et al. 2011) showed dramatic reorientation of the pelvic girdle with respect to the body axis for both chick and frog embryos, the current study is the first to demonstrate that the pelvis also rotates relative to the femur. Unexpectedly, this rotation of the pelvis initiates before the cavity has formed between the femur and pelvis.

It has previously been considered that morphogenesis was a step-by-step process in which certain shape-forming processes could only start after cavitation allowed more physical movements to occur (Pacifici et al. 2005; Khan et al. 2007). Our current study demonstrates instead that morphogenesis is a more continuous process that initiates prior to joint cavitation, a finding in agreement with Roddy et al. (2009). The evidence that shape morphogenesis is significantly advanced prior to cavitation of the joint raises intriguing questions related to movement and joint development. If there is no cavity, what movement, if any, can occur at the joint? Spontaneous movement begins in the chick at day 3 of incubation, and movements of the distal limbs are apparent from day 7 (Mikic et al. 2000), equivalent to HH30/HH31 in our hands. Because cavitation of the hip joint does not occur until HH34 (day 8.5–9), there is a period of time during which limb movements are occurring despite the absence of joint cavities. The interzone, which is highly cellular and low in matrix (Archer et al. 1994), has been shown to be much weaker than the surrounding cartilage in mechanical tests performed by Roddy et al. (2011). Therefore, it is possible that bending at the pre-cavitational joint region is made possible by the decreased rigidity of the interzone region. In axolotl salamanders, some joints never undergo cavitation, but instead the intra-articular region of these joints remains in an interzone-like state (Cosden-Decker et al. 2012). However, articulation of these joints is still possible (Cosden-Decker et al. 2012). While it has previously been suggested that the event of cavitation is precipitated by physical strains induced by bending at the interzone (Ito & Kida, 2000), it is also possible that early shape morphogenesis events could also be influenced by bending at the joint that occurs prior to joint cavitation. Another possibility is that growth-generated strains and pressures, as proposed by Henderson & Carter (2002), could play a role in joint shape morphogenesis. This theory proposes that prior to the onset of movements, the varying patterns of stresses and strains induced by the differential growth of opposing or adjacent developing tissues could act as an important influence on morphogenesis, where local mechanical cues due to such growth-related strains could influence growth rates, tissue differentiation, the direction of growth, and tissue deformation (Henderson & Carter, 2002).

Alternatively, it is possible that morphogenetic events prior to cavitation are intrinsically determined by direct cellular and genetic programs. While an extensive number of studies have investigated the genetic regulation of joint specification and, to a lesser extent, joint cavitation (reviewed in Pacifici et al. 2005; Khan et al. 2007), our understanding of the cellular and genetic regulation of joint shape is sparse in comparison. Pacifici et al. (2005) propose that the shapes of opposing digit epiphyses are molded by preferential growth of the central region of the distal element (to form a convex shape) combined with proliferation at the periphery of the proximal element giving rise to a concave shape at the proximal side. This was based on their finding that the lateral sides of the proximal digit (regions of higher growth) contain a higher number of mitotic cells compared with the central portion, which undergoes less growth (Pacifici et al. 2005). Roddy et al. (2011) also found a relationship between regions of cartilage growth and elevated cell proliferation in the medial condyle of the developing chick knee, suggesting that differential rates of proliferation are responsible for outgrowths that contribute to functional shapes. Investigation of the genetic determinants of joint shape is complicated by the fact that many mutations affecting joint development impact primarily on joint specification (e.g. Noggin: Brunet et al. 1998), a necessary precursor for shape morphogenesis. In human studies, there is a similar lack of understanding of the genetics underlying joint morphogenesis abnormalities, and no genes have yet been identified as contributing to DDH risk for multiple ethnicities (Hogervorst et al. 2012).

The significance of this research is that it provides the first combination of detailed 3D morphological and histological descriptions of early hip joint development in a model system. Three-dimensional imaging has enabled us to show that all three elements of the pelvis are present by HH28, in contrast to what has previously been proposed. Furthermore, we have described dramatic rotation of the pelvis with respect to the femur in a process that starts from HH30. Finally, we have shown for the first time that hip joint shape morphogenesis initiates and progresses to include advanced shape features some days before the cavity forms. These data will form the basis for further characterisation of hip joint morphogenesis, with particular emphasis on hip morphogenesis in the presence of an altered or abnormal mechanical environment as a model system for DDH.

## References

[b1] Abràmoff MD, Magalhães PJ, Ram SJ (2004). Image processing with ImageJ. Biophotonics Int.

[b2] Archer CW, Morrison H, Pitsillides AA (1994). Cellular aspects of the development of diarthrodial joints and articular cartilage. J Anat.

[b3] Bardeen CR, Lewis WH (1901). Development of the limbs, body-wall and back in man. Am J Anat.

[b4] Brunet LJ, McMahon JA, McMahon AP (1998). Noggin, cartilage morphogenesis, and joint formation in the mammalian skeleton. Science.

[b5] Cosden-Decker RS, Bickett MM, Lattermann C (2012). Structural and functional analysis of intra-articular interzone tissue in axolotl salamanders. Osteoarthritis Cartilage.

[b6] DeLaurier A, Schweitzer R, Logan M (2006). Pitx1 determines the morphology of muscle, tendon, and bones of the hindlimb. Dev Biol.

[b7] DeLaurier A, Burton N, Bennett M (2008). The mouse limb anatomy atlas: an interactive 3D tool for studying embryonic limb patterning. BMC Dev Biol.

[b8] Hamburger V, Hamilton HL (1951). A series of normal stages in the development of the chick embryo. Dev Dyn.

[b9] Henderson A (2007). ParaView Guide, A Parallel Visualization Application.

[b10] Henderson J, Carter D (2002). Mechanical induction in limb morphogenesis: the role of growth-generated strains and pressures. Bone.

[b11] Hogervorst T, Eilander W, Fikkers J (2012). Hip ontogenesis: how evolution, genes, and load history shape hip morphotype and cartilotype. Clin Orthop Relat Res.

[b12] Homer C, Baltz R, Hickson G (2000). Clinical practice guideline: early detection of developmental dysplasia of the hip. Committee on Quality Improvement, Subcommittee on Developmental Dysplasia of the Hip. American Academy of Pediatrics. Pediatrics.

[b13] Ito MM, Kida MY (2000). Morphological and biochemical re-evaluation of the process of cavitation in the rat knee joint: cellular and cell strata alterations in the interzone. J Anat.

[b14] Johnson A (1893). On the development of the pelvic girdle and skeleton of the hind-limb in the chick. Quart J Micr Sci.

[b15] Khan I, Redman S, Williams R (2007). The development of synovial joints. Curr Top Dev Biol.

[b16] Lebedinsky N (1913).

[b17] Makovicky PJ, Zanno LE (2011). Theropod Diversity and the Refinement of Avian Characteristics.

[b18] Malashichev Y, Borkhvardt V, Christ B (2005). Differential regulation of avian pelvic girdle development by the limb field ectoderm. Anat Embryol.

[b19] Malashichev Y, Christ B, Pröls F (2008). Avian pelvis originates from lateral plate mesoderm and its development requires signals from both ectoderm and paraxial mesoderm. Cell Tissue Res.

[b20] Mehnert E (1887). Untersuchungen über die Entwicklung des Beckengürtels bei einigen Säugetieren. Morphol Jahrb.

[b21] Mikic B, Johnson TL, Chhabra AB (2000). Differential effects of embryonic immobilization on the development of fibrocartilaginous skeletal elements. J Rehabil Res Dev.

[b22] Nowlan NC, Murphy P, Prendergast PJ (2008). A dynamic pattern of mechanical stimulation promotes ossification in avian embryonic long bones. J Biomech.

[b23] Nowlan NC, Bourdon C, Dumas G (2010). Developing bones are differentially affected by compromised skeletal muscle formation. Bone.

[b24] Osborne AC, Lamb KJ, Lewthwaite JC (2002). Short-term rigid and flaccid paralyses diminish growth of embryonic chick limbs and abrogate joint cavity formation but differentially preserve pre-cavitated joints. J Musculoskelet Neuronal Interact.

[b25] Pacifici M, Koyama E, Iwamoto M (2005). Mechanisms of synovial joint and articular cartilage formation: recent advances, but many lingering mysteries. Birth Defects Res C Embryo Today.

[b26] Pomikal C, Streicher J (2010). 4D-analysis of early pelvic girdle development in the mouse (*Mus musculus*. J Morphol.

[b27] Pomikal C, Blumer R, Streicher J (2011). Four-dimensional analysis of early pelvic girdle development in *Rana temporaria*. J Morphol.

[b28] Roddy KA, Nowlan NC, Prendergast PJ (2009). 3D representation of the developing chick knee joint: a novel approach integrating multiple components. J Anat.

[b29] Roddy KA, Kelly GM, Es van MH (2011). Dynamic patterns of mechanical stimulation co-localise with growth and cell proliferation during morphogenesis in the avian embryonic knee joint. J Biomech.

[b30] Sharpe J, Ahlgren U, Perry P (2002). Optical projection tomography as a tool for 3D microscopy and gene expression studies. Science.

